# Pentadecapeptide BPC 157 Enhances the Growth Hormone Receptor Expression in Tendon Fibroblasts

**DOI:** 10.3390/molecules191119066

**Published:** 2014-11-19

**Authors:** Chung-Hsun Chang, Wen-Chung Tsai, Ya-Hui Hsu, Jong-Hwei Su Pang

**Affiliations:** 1 Graduate Institute of Clinical Medical Sciences, College of Medicine, Chang Gung University, 259 Wen-Hwa 1st Road, Kwei-Shan, Tao-Yuan 333, Taiwan; E-Mails: johnsonchchang@yahoo.com (C.-H.C.); hyh17@cgmh.org.tw (Y.-H.H.); 2 Department of Orthopaedics, National Taiwan University Hospital, Taipei 100, Taiwan; 3 Department of Physical Medicine and Rehabilitation, Chang Gung Memorial Hospital, College of Medicine, Chang Gung University, Kwei-Shan, Tao-Yuan 333, Taiwan; E-Mail: tsaiwc@cgmh.org.tw

**Keywords:** BPC 157, growth hormone receptor, proliferation, PCNA, tendon fibroblast

## Abstract

BPC 157, a pentadecapeptide derived from human gastric juice, has been demonstrated to promote the healing of different tissues, including skin, muscle, bone, ligament and tendon in many animal studies. However, the underlying mechanism has not been fully clarified. The present study aimed to explore the effect of BPC 157 on tendon fibroblasts isolated from Achilles tendon of male Sprague-Dawley rat. From the result of cDNA microarray analysis, growth hormone receptor was revealed as one of the most abundantly up-regulated genes in tendon fibroblasts by BPC 157. BPC 157 dose- and time-dependently increased the expression of growth hormone receptor in tendon fibroblasts at both the mRNA and protein levels as measured by RT/real-time PCR and Western blot, respectively. The addition of growth hormone to BPC 157-treated tendon fibroblasts dose- and time-dependently increased the cell proliferation as determined by MTT assay and PCNA expression by RT/real-time PCR. Janus kinase 2, the downstream signal pathway of growth hormone receptor, was activated time-dependently by stimulating the BPC 157-treated tendon fibroblasts with growth hormone. In conclusion, the BPC 157-induced increase of growth hormone receptor in tendon fibroblasts may potentiate the proliferation-promoting effect of growth hormone and contribute to the healing of tendon.

## 1. Introduction

Tendon injury is one of the most frequent injuries in sport activities. Most tendon injuries are resulted from a tear of tendon fibers due to overuse, aging or accident. The healing of a ruptured tendon is known to be difficult and the repair of completely torn tendon is, therefore, often relies on surgery. Tendon is composed of cells (tendon fibroblasts) and extracellular matrix, which contains mostly type I collagen, type III collagen and glycoproteins. The process of tendon healing is classified into three stages: inflammation, regeneration, and remodeling. In the regeneration stage, tendon fibroblasts migrate into the injured site, proliferate and produce different types of collagens and glycoproteins to form the extracellular matrix. The whole process takes a long time to complete and the healed tendon is always weaker than the tendon before injury [1,2,3]. Therefore, it is important to develop a new therapeutic approach to accelerate the healing and/or improve the strength of tendon.

There are several growth factors involved in the healing process. Insulin-like growth factor, platelet-derived growth factor, transforming growth factor-β, basic fibroblast growth factor and vascular endothelial growth factor are among those mostly mentioned [4,5]. In addition, growth hormone, an anabolic peptide secreted from the anterior pituitary, can also promote tissue regeneration and cell proliferation, especially for the growth of skeletal muscle and bone [6,7]. Growth hormone can also increase the secretion of collagens in different kinds of cultured cells [8,9,10,11]. Through the binding with growth hormone receptor on the cell membrane, the action of growth hormone is mediated directly by the activation of tyrosine kinase or indirectly by induction of insulin-like growth factor [12].

BPC 157 is a pentadecapeptide containing partial sequence of the body protection compound (BPC) isolated from the human gastric juice [13]. It is stable and resistant to hydrolysis or digestion by enzymes. Previous studies have demonstrated the promoting effect of BPC 157 on the healing of different tissues, including skin, mucosa, cornea, muscle, tendon, ligament and bone in animal studies [14,15,16,17,18,19,20,21,22,23,24]. The mechanism by which pentadecapeptide BPC 157 accelerates healing is not clearly understood. It has been suggested to include up-regulation of growth factors [25], proangiogenic effect [26,27], and modulation of nitric oxide (NO) synthesis. BPC 157 may also control functions of collagen fragments that are associated with bone morphogenic proteins. However, none of these functions have been proved experimentally in tendon.

The present study attempted to elucidate the potential mechanism by which BPC 157 works on tendon fibroblasts to improve the tendon healing. From our previous study by cDNA microarray, the expression of growth hormone receptor gene was one of the most up-regulated in tendon fibroblasts treated with BPC 157. We therefore focused this study on the effect of BPC 157 on this gene.

## 2. Results

### 2.1. BPC 157 Induced the Expression of Growth Hormone Receptor in Tendon Fibroblasts

In order to find out the potential mechanism by which BPC 157 improves the healing of tendon, we performed a cDNA microarray in a previous study and the expression of growth hormone receptor gene was one of the most up-regulated in tendon fibroblasts treated with BPC 157 (Table 1).

**Table 1:** Genes up-regulated in tendon fibroblasts by BPC 157.

Gene	Fold Change
HP33	7.71
Potassium voltage gated channel, Shaw-related subfamily, member 2	3.57
Seminal vesicle antigen-like 1	3.47
Retinoblastoma-binding protein 9	3.03
cAMP-regulated guanine nucleotide exchange factor II	2.80
Putative pheromone receptor VN7	2.38
Solute carrier family 26	2.34
Growth hormone receptor	2.29

**Figure 1:**
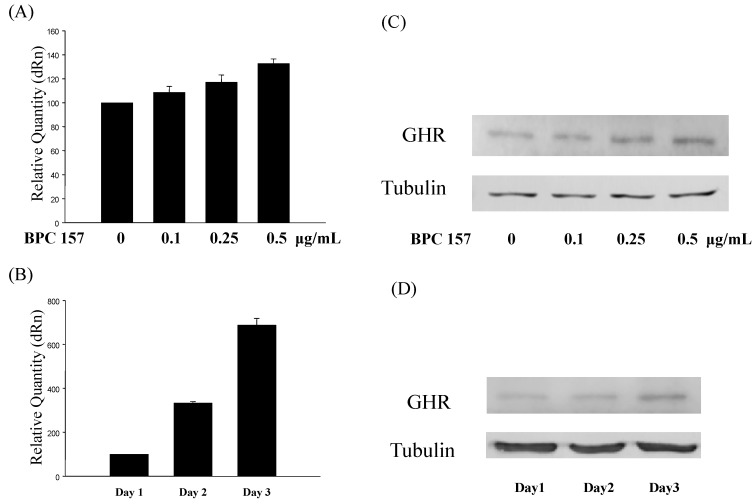
BPC 157 increased the expression of growth hormone receptor in tendon fibroblasts. Tendon fibroblasts at 50%–60% confluency were treated with BPC 157 at concentrations of 0, 0.1, 0.25, 0.5 μg/mL for 24 h (**A** and **C**) or 0.5 μg/mL for one to three days (**B** and **D**). The mRNA (**A** and **B**) and protein (**C** and **D**) expressions of growth hormone receptor were measured by RT/real-time PCR and Western blot analysis, respectively. Experiments were done in triplicate.

To further confirm this result, we treated tendon fibroblasts with BPC 157 at different concentrations (0, 0.1, 0.25 and 0.5 μg/mL) for 24 h and the expression of growth hormone receptor was found to increase in a dose-dependent manner (Figure 1A,C). More interestingly, BPC 157, at the concentration of 0.5 μg/mL, significantly induced the expression of growth hormone receptor in tendon fibroblasts time-dependently from day one to day three (Figure 1B,D). Both the RNA and protein levels were determined by RT/real-time PCR (Figure 1A,B) and Western blotting (Figure 1C,D), respectively. Up to sevenfold increases could be observed at day three.

### 2.2. Growth Hormone Increased the Cell Proliferation of BPC 157-Treated Tendon Fibroblasts

**Figure 2:**
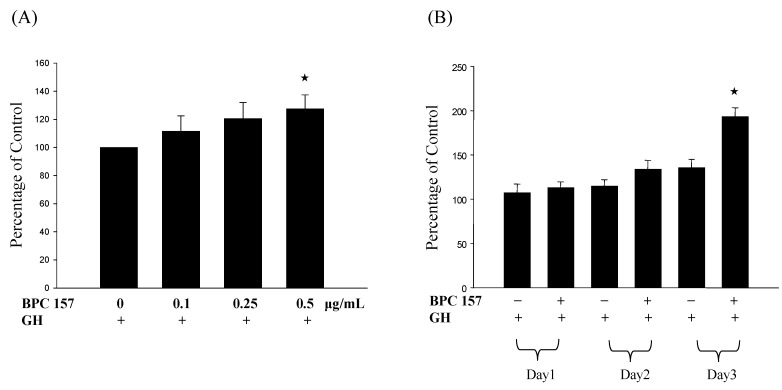
Growth hormone increased the cell number of BPC 157-treated tendon fibroblasts. Tendon fibroblasts at 50%–60% confluency were pretreated with BPC 157 at concentrations of 0, 0.1, 0.25, 0.5 μg/mL for 24 h (**A**) or 0.5 μg/mL for one to three days (**B**). After BPC 157 pretreatment, 0.1 μg/mL growth hormone was added for another 24 h and then MTT assay was performed. Experiments were done in triplicate. The “★” would be applied if there is statistically significant.

**Figure 3:**
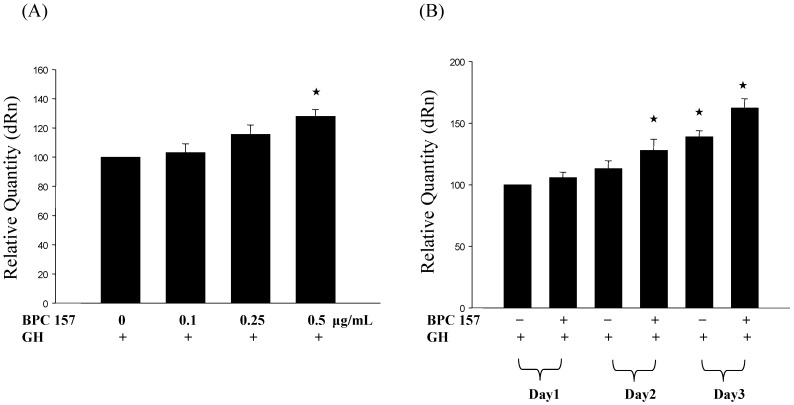
Growth hormone increased the PCNA gene expression in BPC 157-treated tendon fibroblasts. Tendon fibroblasts at 50%–60% confluency were pretreated with BPC 157 at concentrations of 0, 0.1, 0.25, 0.5 μg/mL for 24 h (**A**) or 0.5 μg/mL for one to three days (**B**). After BPC 157 pretreatment, 0.1 μg/mL growth hormone was added for another 24 h and then the PCNA gene expression was analyzed by RT/real-time PCR. Experiments were done in triplicate. The “★” would be applied if there is statistically significant.

Since growth hormone receptor, after the binding of growth hormone, can trigger the activation of signal pathway and eventually lead to the increase of cell proliferation [28]. We, therefore, tested the effect of adding growth hormone to BPC 157-treated tendon fibroblasts. Tendon fibroblasts were treated with BPC 157 at different concentrations (0, 0.1, 0.25 and 0.5 μg/mL) for first 24 h and 0.1 μg/mL growth hormone was added for another 24 h and cell viability was determined by MTT assay. Results in Figure 2A demonstrated a dose-dependent increase of viable cells after the addition of growth hormone. Similarly, the number of viable cells after the addition of 0.1 μg/mL growth hormone to BPC 157-treated cells for one to three days significantly increased in a time-dependent manner (Figure 2B), proving that BPC 157 pretreatment could potentiate the effect of growth hormone on increasing the number of viable cells. To further demonstrate the effect of BPC 157 and growth hormone on the cell proliferation of tendon fibroblasts, we analyzed the expression of cell proliferation marker, proliferating cell nuclear antigen (PCNA) in tendon fibroblasts at the RNA expression level by RT/real-time PCR. Results in Figure 3A,B revealed a similar pattern of PCNA induction in tendon fibroblasts by BPC 157 and growth hormone that was correlated very well with the increase of viable cell number.

### 2.3. Growth Hormone Activated the Janus Kinase 2 Gene (Jak2) in BPC 157-Treated Tendon Fibroblasts

**Figure 4:**
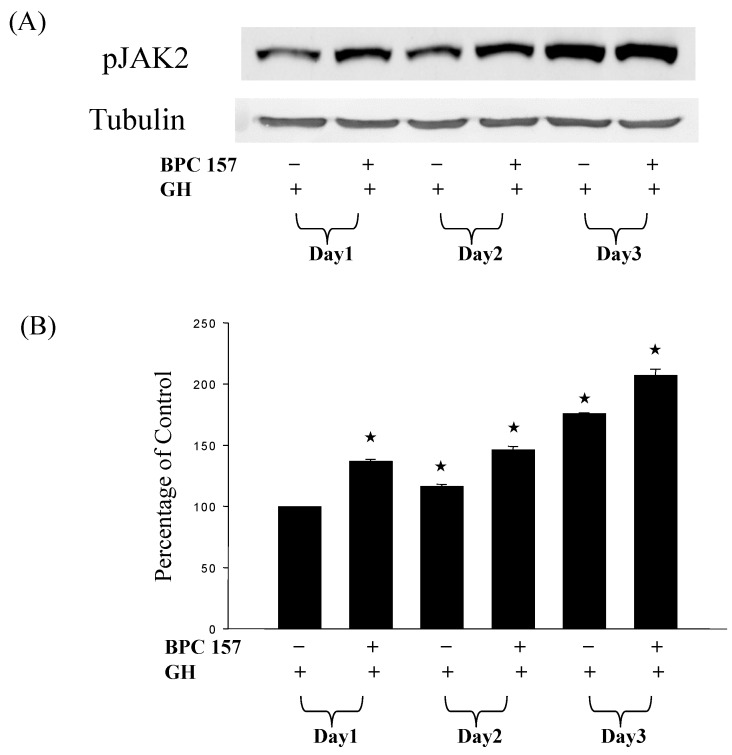
Growth hormone activated more JAK2 proteins in BPC 157-treated tendon fibroblasts. Tendon fibroblasts at 50%–60% confluency were pretreated with BPC 157 at concentration of 0.5 μg/mL for one to three days. After BPC 157 pretreatment, 0.1 μg/mL growth hormone was added for another 24 h and then total JAK2 (which was not shown) and activation of JAK2 which was determined by the level of phosphorylated JAK2 were detected by Western blot analysis (**A**). Experiments were done in triplicate. The levels of phosphorylated JAK2 were calculated by direct densitomeric analysis of the blot (**B**). The “★” would be applied if there is statistically significant.

Through the JAK-STAT signaling pathway, growth hormone can exert the growth-stimulating effects on different tissues. Therefore, we investigated the effect of growth hormone on the activation of Jak2 in BPC 157-treated tendon fibroblasts. As shown in Figure 4, the addition of 1 μg/mL growth hormone did activate Jak2 signal pathway in tendon fibroblasts pretreated with 0.5 μg/mL BPC 157. Longer treatment with BPC 157, the level of phosphorylated but not the total amount of Jak2 was found to be higher by the stimulation with growth hormone.

## 3. Discussion

Body protection compound (BPC, M.W. 40,000) was first discovered and isolated in human gastric juice and later a stable 15 amino acid fragment (Gly Glu Pro Pro Pro Gly Lys Pro Ala Asp Asp Ala Gly Leu Val, M.W. 1419, called BPC 157) with apparently no sequence homology to other known peptides, was found to be essential for BPC’s activity [23]. Although the detailed mechanism is poorly understood, BPC 157 appears to be beneficial to almost all organ systems in many species when very low dosages (mostly ng/kg to μg/kg range) after ip, ig, and intramucosal (local) application are used and no side effect or toxicity is found. Except the effects on various gastrointestinal lesions, the healing effects of BPC 157 have also been reported on pancreas, liver injuries, endothelium, heart damage and pseudoarthrosis. Interestingly, the healing of transected rat Achilles tendon could be accelerated by BPC 157 and even the early functional recovery of tendon to bone after Achilles detachment was found to be promoted by BPC 157 [19].

Our previous study has shown that BPC 157 actually has no direct effect on promoting the proliferation or the expression of PCNA gene in tendon fibroblasts, although it enhances the migratory ability of tendon fibroblasts [29]. The healing of the injured tendon involves a lot of complex pathways. It progresses through overlapping stages of inflammation, regeneration and remodeling. The process of regeneration is believed to occur either extrinsically by infiltration of external cells, intrinsically by tendon fibroblast proliferation, or both. Migration and proliferation of cells seem to be fundamental for tendon healing. If BPC 157 does not directly promote the proliferation of tendon fibroblasts, what could be the potential mechanism underlying its healing effect? We therefore screened the effect of BPC 157 on tendon fibroblasts by cDNA microarray analysis in our previous study. Several genes were up-regulated by BPC 157 and growth hormone receptor is one of the top five (Table 1).

The present study, for the first time, demonstrated that the expressions of growth hormone receptor at both the mRNA and protein levels in tendon fibroblasts were increased by BPC 157. The promoting effect of BPC 157 on the expression of growth hormone receptor was further found to be more significant up to three days after the treatment. In combination with the addition of growth hormone, the proliferation and the PCNA expression of tendon fibroblasts were both enhanced in tendon fibroblasts. The addition of growth hormone was confirmed to activate the phosphorylation but not the total amount of JAK2, the downstream signal pathway of growth hormone receptor, to a more significant level in tendon fibroblasts with longer treatment with BPC 157, providing a new potential mechanism for the healing effect of BPC 157.

Growth hormone is a peptide hormone that stimulates growth, cell reproduction and regeneration of chondrocytes and osteoblasts in humans and other animals [30]. Growth hormone is used as a prescription drug in medicine to treat children’s growth disorders and adult growth hormone deficiency. It is known to be capable of increasing the protein production and muscle mass [12]. Growth hormone can also increase matrix collage synthesis in human skeletal muscles and tendons. After binding with the surface receptor on target cells, growth hormone can activate the JAK-STAT signaling pathway and stimulate the production of insulin-like growth factor 1 (IGF-1), which is known to exert a growth-stimulating effect on different tissues. Interestingly, a recent study showed that the strength of the lower part of healthy men has also been increased by growth hormone therapy and a clinical trial is now undergoing for older population. Growth hormone was also reported to have effects of migration and adhesion of different cells. Taub *et al.* reported that through chemokinetic effect, growth hormone could significantly induce the migration of resting and activated human T cells [31]. Savino also reported that growth hormone has a role in migration of developing thymocytes [32]. Lee *et al.* reported that through IGF-1, growth hormone might activate fibroblast proliferation and keratinocyte migration [33]. These effects all contribute to the process of tendon healing.

Our results showed that, in the presence of same amount of growth hormone, pretreatment with BPC 157 can no doubt enhance the effect of growth hormone in a dose- and time-dependent manner. More importantly, the effect of BPC 157 can last for at least three days in the cultured tendon fibroblasts, confirming the stability of this pentadecapeptide and only low dose is required for sustained effect. However, we understood that the experimental condition using* in vitro* culture of tendon fibroblasts could not mimic the real environment of tendon. During* in vivo* healing course, other cells, such as leukocytes and stem cells, may also interact with each other and contribute to this complicated process. It is possible that the healing accelerating effect of BPC 157 may act on other cells and exert an indirect effect on promoting the proliferation of tendon fibroblasts.

## 4. Experimental Section

### 4.1. Primary Culture of Tendon Fibroblasts from Rat

This study has been approved by the Institutional Animal Care and Use Committee before the procedures were performed. Male Sprague-Dawley rats, weighing 200 to 250 gm, were used as the source of tendon fibroblasts in this study. Achilles tendons were first harvested from rats by aseptic procedures. Each tendon was chopped into pieces at size about 1.5 to 2.0 mm^3^ and separately put into six-well culture plates. Then 3 mL culture medium made of Dulbecco’s modified Eagle’s medium with 10% FBS, 100 U/mL penicillin, and 100 mg/mL streptomycin was added to each well and maintained at 37 °C in a humidified atmosphere of 95% air and 5% CO_2_. After migrating out from the explants, tendon fibroblasts started to grow rapidly. After reaching confluency, the cells were subcultured by trypsinization at a 1:3 dilution ratio. Tendon fibroblasts between passages 2 and 4, having proper growth rate and normal fibroblast shape, were used in the following experiments. Each experiment was repeated three times using tendon fibroblasts isolated from a different rat. 

### 4.2. BPC 157 Treatment

Pentadecapeptide BPC 157 (GEPPPGKPADDAGLV, M.W. 1419) was synthesized and purchased from Kelowna International Scientific Inc., Taipei, Taiwan. BPC 157 was added to cells at the concentrations of 0 (control group), 0.1, 0.25 and 0.5 μg/mL. After incubation at 37 °C in a humidified atmosphere of 5% CO_2_/95% air for 1, 2 and 3 days, cells were collected for analysis of the expression of growth hormone receptors by RT PCR and Western blotting. 

### 4.3. Real-Time PCR

Total RNA was extracted from cells by acid guanidinium thiocyanate-phenol-chloroform extraction method, and complementary (c)DNA was synthesized using 1 μg total RNA in a 20 μL RT reaction mix containing 0.5 μg/μL of random primers, 0.1 mM dNTP, 0.1 M DTT and 5× first strand buffer. Real-time PCR was performed using an SYBR Green I technology and MxPro- Mx3000P QPCR machine (Stratagene, CA, USA), and a master mix was prepared with Smart Quant Green Master Mix with dUTP & ROX Kit (Protech, Taipei, Taiwan). Relative gene expressions between experimental groups were determined using MxPro software (Stratagene, CA, USA) and GAPDH was used as an internal control. All real-time PCRs were performed in triplicate, and changes in gene expressions were reported as multiples of increases relative to the controls. The following primers were used: GAPDH: 5'-GAGGGGCCATCCACAGTCTT-3' (forward) and 5'-TTCATTGACCTCAACTACAT-3' (reverse), GHR: 5'-GATGTTCTGAAGGGATGG-3' (forward) and 5'-GTGGGACTGATGTTGACC-3' (reverse) and PCNA: 5'-GAAGCACCAAATCAAGAG-3' (forward) and 5'-CATCTCCAATATGGCTAA-3' (reverse).

### 4.4. Western Blot Analysis

Cell extracts were prepared in lysis buffer containing Tris-HCl, pH 7.5, 150 mM NaCl, 1 mM EDTA, 2 mM DTT, 2 mM PMSF and 1% Triton X-100 followed by ultrasonication method. Protein concentration of the cell extracts was determined by Bradford assay (Bio-Rad Laboratories, CA, USA). Samples with same amount of proteins were then separated by 12% sodium dodecyl sulfate (SDS) polyacrylamide gel electrophoresis and transferred onto a PVDF membrane. Membrane was incubated at room temperature in blocking solution (1% bovine serum albumin, 1% goat serum in 1×PBS) for 1 h, followed by incubation in blocking solution containing appropriate dilution of primary antibody for growth hormone receptor (R&D System, MN, USA) or phospho-Jak2 (Tyr1007/1008, Cell Signaling Technology, Inc. Beverly, MA, USA) for two hours. After washed three times in 1×PBS, the membrane was then incubated in 1×PBS containing goat anti-mouse IgG conjugated with horseradish peroxidase (Sigma, St. Louis, MO, USA) for 1 h. Membranes were washed three times in 1×PBS and positive signals were developed with enhanced chemiluminescence kit (Amershan Pharmacia Biotech, Little Chalfont Buckinghamshire, England).

### 4.5. MTT Assay

1 × 10^5^ tendon fibroblasts were seeded in each well of 24-well culture plate, which contained culture medium made of 0.5 mL of DMEM, 10% FBS, 100 U/mL penicillin, and 100 mg/mL streptomycin in each well. BPC 157 was added into each well at the concentration of 0 (control group), 0.1, 0.25 and 0.5 μg/mL for 1, 2, and 3 days at 37 °C in a humidified atmosphere of 5% CO_2_/95% air. One day after the addition of growth hormone to the BPC 157-treated tendon fibroblasts, cells were washed once with 1xPBS, followed by adding 1 mL DMEM containing 0.05 mg/mL 3-[4,5-dimethylthiazol-2-yl]-2, 5-diphenyltetrazolium bromide (MTT). After incubation at 37 °C for 1 h, the media were removed and formazan crystals in the cells were dissolved in 1 mL DMSO and processed for OD reading at 570 nm using a spectrophotometer. The experiments were performed in triplicate.

### 4.6. Statistical Analysis

All data were expressed as mean ± SEM. Quantification of phosphorylated Jak2 and tubulin expressions were performed by calculating the band density using 1D Digital Analysis Software (Kodak Digital ScienceTM, Eastman Kodak Company, Rochester, NY, USA). Comparisons between control group and experimental group were performed using Student’s *t* test. The level of statistical significance was set at a *p* value of 0.05. The “★” would be applied if there is statistically significant.

## 5. Conclusions

This study demonstrated the promoting effect of BPC 157 on tissue healing is potentially associated with the increased expression of growth hormone receptor in tendon fibroblasts. This finding also suggests a different way to promote the tissue healing by increasing the expression of growth hormone receptor to promote the beneficial effect of growth hormone in terms of enhanced proliferation. In addition, the amount of growth hormone used can theoretically be reduced and also the cost of therapy. BPC 157 may play an important role in promoting tendon healing and potential clinical usage in the future is expected.
